# National genomic surveillance of carbapenemase-producing *Escherichia coli* isolates in Slovenia, January 1st–December 31st 2023

**DOI:** 10.3389/fpubh.2026.1924446

**Published:** 2026-09-16

**Authors:** Mateja Pirs, Urška Kramar, Tina Triglav, Katja Strašek Smrdel, Andraž Celar Šturm, Lara Bezjak, Sandra Janezic

**Affiliations:** 1 Institute of Microbiology and Immunology, Faculty of Medicine, University of Ljubljana, Ljubljana, Slovenia; 2 National Laboratory for Health, Environment and Food, Centre for Medical Microbiology, Maribor, Slovenia

**Keywords:** carbapenemase, *Escherichia coli*, plasmid, population structure, WGS

## Abstract

**Introduction:**

The incidence of carbapenemase-producing (CP) *Escherichia coli* has increased worldwide, with the first reports in Europe dating back to the early 2000s. As part of the European Genomic Reference Laboratory Capacity Building (EURGen-RefLabCap) project, Slovenia conducted its first national genomic surveillance study of CP–*E. coli*. The aim was to characterize CP–*E. coli* isolates recovered from Slovenian patients between 1 January and 31 December 2023 using whole-genome sequencing (WGS), providing a national snapshot of the population structure and circulating strains.

**Methods:**

WGS was performed using short-read sequencing. Bioinformatic analyses included identification of antimicrobial resistance (AMR) determinants, multilocus sequence typing (MLST), phylogroup typing, core genome multilocus sequence typing (cgMLST), assessment of genetic relatedness, and plasmid reconstruction using MOB-suite.

**Results:**

A total of 108 CP–*E. coli* isolates (108 patients) were included. Most isolates originated from surveillance samples (89.8%), 30.6% were detected more than 48 h after hospital admission. OXA-48-like carbapenemases predominated (70.4%), followed by metallo-β-lactamases (29.6%), with OXA-48 (44.4%), OXA-244 (22.2%), and VIM-1 (18.5%) being the most common enzymes. Despite carbapenemase production, 69.4% of isolates remained phenotypically susceptible to carbapenems. WGS revealed substantial genetic diversity, with 67 sequence types (STs) identified among 108 isolates. The most common lineages were ST10 (13.9%), ST38 (12.0%), ST5295 (9.3%), and ST69 (6.5%). Phylogroup A was the most prevalent, followed by D, B2, and B1. cgMLST analysis identified six transmission clusters comprising 26 isolates (24.1%), the remaining isolates were sporadic. Plasmid analysis based on short-read sequencing demonstrated extensive horizontal dissemination of carbapenemase genes, with blaOXA-48 predominantly associated with IncL/M plasmids and blaVIM-1 with IncA plasmids. Closely related plasmids were detected across multiple genetically distinct STs, indicating widespread plasmid-mediated transmission.

**Discussion:**

CP–*E. coli* in Slovenia is characterized by a rising burden, high genetic diversity, and a predominance of OXA-48-like carbapenemases. While limited clonal transmission was observed, the widespread distribution of closely related carbapenemase-carrying plasmids across diverse sequence types indicates that horizontal plasmid transfer is the principal driver of dissemination. These findings highlight the value of the national genomic surveillance for monitoring both clonal spread and plasmid-mediated transmission of carbapenem resistance.

## 1 Introduction

*Escherichia coli* is the leading cause of bacterial infectious diseases both in community and hospital settings, and the leading cause of deaths attributable to antimicrobial resistance (AMR) among bacterial pathogens (1). In the late 1990s extended-spectrum beta lactamases (ESBLs) began to spread widely among *E. coli*, followed by the global dissemination of high-risk clones resistant to third-generation cephalosporins and frequently also to fluoroquinolones [e.g., sequence type (ST) ST131] (2, 3). This has been followed by a slow increase in resistance to carbapenems and the spread of carbapenemase producing *E. coli* (CP–*E. coli*) (4, 5). The earliest reports of CP–*E. coli* in Europe date from the 2000s (6–8).

The increasing incidence of CP–*E. coli* has been reported worldwide particularly over the past decade. This expansion is frequently associated with the dissemination of a limited number of successful high-risk clonal lineages, notably sequence types (ST) ST131, ST410, ST167, and ST405, associated with carbapenemase genes such as *bla*_NDM_, *bla*_OXA − 48 − like_, and *bla*_KPC_ (9, 10). In a recently published study, Huang et al. reported that the spread of CP–*E. coli* has been associated with shifts in the prevalence of *bla*_NDM − 5_, *bla*_KPC − 2_, and *bla*_OXA − 48 − like_ carbapenemase genes, alongside the dissemination of IncFI, IncFII, and IncI plasmids. Intercontinental dissemination of major carbapenem-resistant *E. coli* clones has been documented, with prominent transmission networks involving ST131 across the United Kingdom, Italy, the United States, and China; ST167 across India, France, Egypt, and the United States; and ST410 across Thailand, Israel, the United Kingdom, France, and the United States (11).

The first survey to assess the capacity of European countries to detect NDM carbapenemase producers, conducted by the European Centre for Disease Prevention and Control (ECDC), took place in 2011 (12). This was followed by three structured European surveys on carbapenemase-producing Enterobacterales (CPE). The first was the European Survey of Carbapenemase-Producing Enterobacteriaceae (EuSCAPE) in 2013–2014, followed by the carbapenem- and/or colistin-resistant Enterobacterales (CCRE) survey in 2019 and the Survey of carbapenem-resistant Enterobacterales 2025 (CRE25) in 2025 (9, 13, 14). The results of both the 2013–2014 EuSCAPE and the 2019 CCRE surveys show that CPE has been slower to emerge and spread in *E. coli* than in *Klebsiella pneumoniae* in Europe. The ratio of CP–*K. pneumoniae* to CP–*E. coli* among hospitalized patients was approximately 11:1 (810 CP–*K. pneumoniae* vs. 77 CP–*E. coli*) in the EuSCAPE study but decreased to 8:1 in the CCRE study (1398 CP–*K. pneumoniae* vs. 182 CP–*E. coli*) (9, 14).

As of 2026, no formal national surveillance system for CPE exists in Slovenia. Available national data are derived from surveillance activities and studies performed by the Slovenian Society for Clinical Microbiology and Hospital Infection and the Slovenian National Antimicrobial Committee (NAC). To assess temporal trends in CP–*E. coli*, participating microbiology laboratories analyzed annual regional data collected between 2017 and 2025, which were subsequently aggregated at the national level. Only the first CP–*E. coli* isolate per patient per calendar year was included, with analyses stratified by specimen type (clinical or surveillance). Between 2017 and 2025, the number of patients with CP–*E. coli* identified from clinical specimens increased from 2 to 39, representing a 19.5-fold increase (15).

The European Genomic Reference Laboratory Capacity Building (EURGen-RefLabCap) project was an EU-funded initiative (2020–2024) coordinated by the ECDC in collaboration with the Technical University of Denmark and Statens Serum Institute. Its objective was to strengthen genomic surveillance and reference laboratory capacity for AMR across 37 European countries (16). Slovenia participated as one of the thirteen priority countries selected through a mapping exercise based on their relatively greater need for targeted support to strengthen National Reference Laboratory (NRL) capacity or National Expert Laboratory (NEL) capacity for the surveillance of priority antimicrobial-resistant pathogens. Slovenia has AMR NRL capacity available at two institutions with NELs. As one of the EURGen-RefLabCap priority countries, Slovenia received support to conduct a pilot genomic surveillance study, applying newly acquired knowledge and technical skills in whole genome sequencing (WGS) and bioinformatics analysis, and generating the first truly national WGS genomic data on AMR in Slovenia for a priority pathogen (*E. coli* or *K. pneumoniae*).

The aim of the Slovenian EURGen-RefLabCap pilot genomic surveillance study was to perform the first national study on CP–*E. coli* and sequence all non-duplicate CP–*E. coli* isolates recovered from Slovenian patients between January 1st and December 31st 2023, thereby providing a snapshot of the population dynamics of circulating strains.

## 2 Materials and methods

### 2.1 Participating laboratories

Slovenia has no AMR NRL, however NRL capacity is available at two institutions with NEL at the Institute for Microbiology and Immunology, Faculty of Medicine, University of Ljubljana (IMI) and at the National Laboratory for Health, Environment and Food (NLZOH).

The IMI NEL processed isolates collected by IMI from two Slovenian regions, as well as isolates received from three hospital microbiology laboratories serving three additional regions. The NLZOH NEL processed samples collected from eight Slovenian regions, thereby ensuring coverage of healthcare facilities across the entire country.

### 2.2 Isolate collection and isolate inclusion criteria

Bacterial isolates were collected from routine clinical and surveillance samples. Clinical samples were cultured in accordance with standard microbiological procedures appropriate for the specimen type, using conventional culture media, including blood agar, MacConkey agar, and chromogenic urinary agar at 35–37°C for 18–24 h. Surveillance samples were cultivated directly on selective chromogenic media (e.g., mSuperCARBA agar (CHROMagar, Paris, France) or chromID CARBA SMART (CARB/OXA) agar (bioMérieux, Marcy-l'Étoile, France), according to the routine practice of each participating laboratory), followed by an enrichment step using Tryptic soy broth with vancomycin and subsequent plating onto selective chromogenic media.

Bacterial isolates were identified using matrix-assisted laser desorption/ionization time-of-flight (MALDI-TOF) mass spectrometry. Antimicrobial susceptibility testing was performed according to EUCAST guidelines. A CPE isolate was defined as an isolate demonstrating carbapenemase production based on EUCAST screening criteria and positive phenotypic and/or molecular confirmatory testing performed in accordance with EUCAST recommendations and Slovenian NAC guidelines (17, 18). Phenotypic confirmation was performed at the local laboratory level and included colorimetric tests (e.g., *in house* Carba NP or β CARBA Test (Bio-Rad Laboratories, Marnes-la-Coquette, France), according to the routine practice of each participating laboratory), carbapenem inactivation method (CIM)-based assays, and the Carba-5 test (NG-Biotech, Guipry-Messac, France) (18–20).

A single CP–*E. coli* isolate harboring a specific carbapenemase gene was included per patient, regardless of specimen type (clinical or surveillance). When multiple isolates carrying the same carbapenemase were recovered from a single patient, only one isolate was included; clinical isolates were prioritized over surveillance isolates. The isolates were stored at −80°C and revived for WGS.

#### 2.2.1 Isolate/patient metadata

Isolates were obtained mainly from patients in secondary hospitals and both tertiary care centers, specialized institutions (e.g., Oncology Institute and University Rehabilitation Institute), as well as in primary healthcare settings, and long-term care facilities (LTCFs). Basic anonymized metadata were collected for each sample, including the date of receipt by the laboratory, healthcare setting (primary care, hospital, or LTCF), patient age and sex, sample type (clinical or surveillance), time since hospital admission for hospitalized patients (≤48 h or >48 h), bacterial species, susceptibility to carbapenems (meropenem), and the detected carbapenemase.

### 2.3 DNA extraction

Genomic DNA was extracted using the DNeasy Blood & Tissue Kit or QIAamp DNA Mini Kit (Qiagen Ltd, West Sussex, United Kingdom), according to the manufacturer's protocol for Gram-negative bacteria. The extracted DNA was stored at −80 °C until further use. DNA concentrations were quantified using the Qubit 3.0 fluorometer with the Qubit 1× dsDNA HS Assay Kit (Thermo Fisher Scientific, Waltham, MA, USA).

### 2.4 Whole genome sequencing

WGS was performed on the NextSeq 550 or 2000 platform (Illumina, San Diego, California, USA) using paired-end reads chemistry (2 × 150 or 2 × 300 cycles), with a minimum target genome coverage of at least 100×. Genomic DNA libraries were prepared using the Illumina DNA Prep workflow (Illumina, San Diego, California, USA) or the NEBNext Ultra II FS DNA Library Prep Kit for Illumina (New England Biolabs, UK) according to the manufacturer's instructions.

#### 2.4.1 Raw data pre-processing

Adapter sequences and low-quality reads were removed from the raw sequencing data using Trimmomatic v0.39 (21). The quality of raw and trimmed reads was assessed using FastQC v0.11.9 (22). Trimmed sequencing reads were used for species confirmation with KmerFinder v3.0.2.

#### 2.4.2 De novo assembly and QC

*De novo* assembly of raw reads into contigs was performed using SPAdes v3.13.1 with default parameters (23). Quast v5.2.0 was used for the quality assessment of the assemblies (24) and genome coverage was estimated using Samtools v1.10 (25, 26). Assemblies were considered to be of high (adequate) quality if they met the following thresholds: N50 greater than 20,000, fewer than 500 contigs, and genome size within 10% of the reference genome (27). QC metrics for whole-genome sequencing data and genome assemblies are presented in Supplementary Table S1.

#### 2.4.3 Analysis of antimicrobial resistance

AMRFinderPlus v4.2.7 (database version 2026-05-15.1) was used to detect AMR genes, using assembled sequences as input, with minimum identity and coverage thresholds set to 80% and 50%, respectively (28).

#### 2.4.4 Sequence typing

Sequence types (STs) were assigned according to the Warwick seven-locus MLST typing scheme (29) using MBioSeq software v12.0.5 (Bruker, Bremen Germany, formerly SeqSphere+ Ridom GmbH, Münster, Germany).

#### 2.4.5 cgMLST analysis

Core genes present in ≥95% of isolates were identified using the MBioSeq *E. coli* cgMLST scheme with default settings (30). Isolates differing by ≤ 10 alleles were considered closely related and assigned to the same cgMLST cluster (27).

#### 2.4.6 Phylogroup typing

Phylogenetic classification of isolates was performed *in silico* using ClermontTyping v.24.02 (31).

### 2.5 Plasmid analysis

Plasmid analysis was performed on draft genome assemblies in FASTA format using MOB-suite v3.1.8 (32, 33), which enables plasmid identification, reconstruction, classification, and replicon typing. Plasmids were grouped using the primary MOB-suite cluster classification, which is based on overall sequence similarity and defined by a MASH distance threshold of 0.06.

Single nucleotide polymorphisms (SNPs) among plasmid sequences were identified using Snippy v4.6.0 (34). Within each primary MOB-suite cluster, the largest plasmid was selected as the reference to maximize coverage, and all remaining plasmids were aligned against it. Alignment statistics for plasmids assigned to primary MOB-suite cluster together with summary statistics for the core alignment is presented in Supplementary Table S2. Core SNP alignments comprising SNP positions shared by all plasmids within the cluster were generated for downstream analyses. Putative recombinant regions were identified and removed using Gubbins v3.1.0 (35), and SNP sites were subsequently extracted from the filtered alignments using SNP-sites v2.5.1 (36).

Comparative plasmid visualization was performed using BLAST Ring Image Generator (BRIG) v1.0.0 (37), applying a minimum identity threshold of 70% and 100% alignment coverage. For each primary MOB-suite cluster, the most frequently identified complete reference plasmid was selected based on MOB-suite database matches, and the corresponding sequence was retrieved from NCBI. The reference plasmids used were CP018717 for cluster AA002, MH220284 for AA621, CP045282 for AA001, CP026476 for AA038, CP026661 for AA739. As closest NCBI reference for cluster AC288 (NC_019984) did not contain the *bla*NDM-5 gene, plasmid SI_46 was used as reference. External reference plasmids were used because fragmented plasmid assemblies cannot be reliably visualized in BRIG; therefore, genomic regions absent from the selected reference plasmid are not represented in the comparison. The countries of origin of the NCBI reference plasmids are listed in Supplementary Table S3.

## 3 Results

### 3.1 Demographic data

CP–*E. coli* cases occurred throughout 2023 at a generally low level, with between 0 and 5 cases reported per week. Weekly case numbers fluctuated over time; no clear outbreak peak was observed (Figure 1). In total 108 isolates from 108 patients were included in the study; 61 (56.5%) were male, and 47 (43.5%) were female. The median age was 70 years (range 21 to 91 years). Most of the isolates were collected from surveillance rectal samples (97/108, 89.8%), followed by urine (7/108, 6.5%), blood (2/108, 1.9%), and one isolate (1/108, 0.9%) each from a wound and a lower respiratory tract sample.

**Figure 1:**
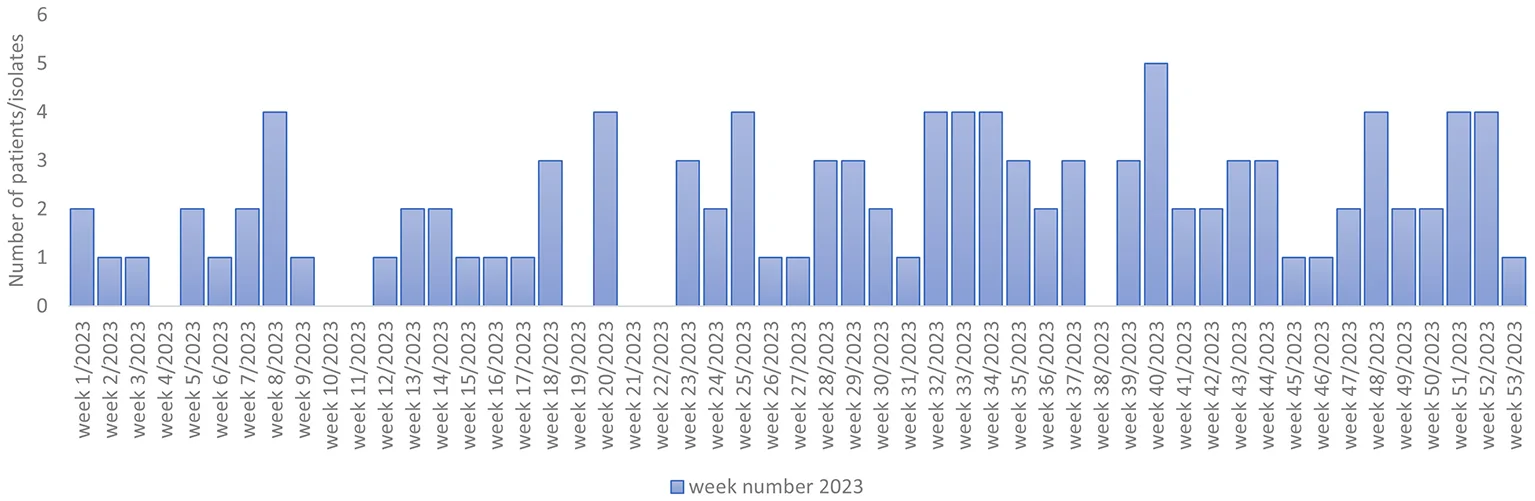
The epidemic curve showing low-level occurrence of CP–*E. coli* throughout 2023.

Fifty isolates (46.3%) were recovered from patients within 48 h of hospital admission, whereas 33 (30.6%) were obtained more than 48 h after admission. One isolate (0.9%) originated from a patient in a long-term care facility. Eleven patients (10.2%) were managed at primary care level, with no information available regarding subsequent hospital admission. The remaining 13 patients (12.0%) were hospitalized, but the date of admission was unavailable.

### 3.2 Carbapenemases

The majority (75/108, 69.4%) of CP–*E. coli* isolates were phenotypically susceptible to carbapenems. Serine carbapenemases were present in 76/108 (70.4%) of all isolates, with OXA-48 being most prevalent (48/108, 44.4 %), followed by OXA-244 (24/108, 22.2%), OXA-181 (3/108, 2.8%) and KPC-3 (1/108, 0.9%). Metallo-beta lactamases were identified in 32/108 (29.6%) isolates, predominantly VIM-1 (20/108, 18.5%), followed by NDM-5 (5/108, 4.6%), and NDM-1 (4/108, 3.7%). Three isolates harbored both an OXA-carbapenemase and a metallo-beta lactamase (Table 1).

**Table 1:** Carbapenemase genes and carbapenem susceptibility in CP–*E. coli*.

Carbapenemase gene	meropenem S/I	meropenem R	All isolates
	**75 (69.4%)**	**33 (30.6%)**	**108**
*bla*_KPC − 3_	1 (1.3%)	0	1 (0.9%)
*bla*_OXA − 48_	38 (50.7%)	10 (30.3%)	48 (44.4%)
*bla*_OXA − 181_	3 (4%)	(0%)	3 (2.8%)
*bla*_OXA − 244_	22 (29.3%)	2 (6.1%)	24 (22.2%)
*bla*_NDM − 1_	2 (2.7%)	2 (6.1%)	4 (3.7%)
*bla*_NDM − 5_	1 (1.3%)	4 (12.1%)	5 (4.6%)
*bla*_VIM − 1_	8 (10.7%)	12 (36.4%)	20 (18.5%)
*bla*_VIM − 1/_ *bla*_OXA − 48_	0	1 (3%)	1 (0.9%)
*bla*_NDM − 5/_ *bla*_OXA − 48_	0	1 (3%)	1 (0.9%)
*bla*_NDM − 5/_ *bla*_OXA − 181_	0	1 (3%)	1 (0.9%)

Among isolates carrying OXA-type carbapenemases, ESBL genes were frequently detected (35/75, 46.7%). One isolate (1.3%) harbored a plasmid-mediated AmpC gene in the absence of an ESBL, while two isolates (2.6%) co-harbored both ESBL and plasmid-mediated AmpC genes. Both ESBL and AmpC beta lactamases were also found in isolates with metallo-beta lactamases (Table 2). Select antimicrobial resistance determinants detected in CP–*E. coli* isolates using AMRFinderPlus are presented in Supplementary Table S4.

**Table 2:** The presence of ESBL or AmpC genes among CP–*E. coli* isolates.

Carbapenemase gene	ESBL or AmpC betalactamase gene	No (%) per carbapenemase isolates
*bla*_KPC − 3_	None	1 (100%)
*bla*_OXA − 48_	None	27 (56.3%)
	*bla*_CMY − 4_ (AmpC)/ *bla*_CTX − M−15_ (ESBL)	1 (2.1%)
	*bla*_CTX − M−14_ (ESBL)	13 (27.1%)
	*bla*_CTX − M−15_ (ESBL)	4 (8.3%)
	*bla*_CTX − M−3_ (ESBL)	1 (2.1%)
	*bla*_CTX − M−9_ (ESBL)	1 (2.1%)
	*bla*_DHA − 1_ (AmpC)	1 (2.1%)
*bla*_OXA − 244_	None	8 (33.3%)
	*bla*_CTX − M−14_ (ESBL)	1 (4.2%)
	*bla*_CTX − M−15_ (ESBL)	3 (12.5%)
	*bla*_CTX − M−231_ (ESBL)	8 (33.3%)
	*bla*_CTX − M−27_ (ESBL)	4 (16.7%)
*bla*_OXA − 181_	None	2 (100%)
	*bla*_CMY − 2_ (AmpC)/ *bla*_CTX − M−15_ (ESBL)	1 (50%)
*bla*_NDM − 1_	None	2 (50%)
	*bla*_CMY − 16_ (AmpC)	1 (25%)
	*bla*_CTX − M−15_ (ESBL)	1 (25%)
*bla*_NDM − 5_	*bla*_CMY − 145_ (AmpC)	1 (20%)
	*bla*_CTX − M−15_ (ESBL)	2 (40%)
	*bla*_CTX − M−15_ (ESBL)/ *blaDHA-1* (AmpC)	2 (40%)
*bla*_VIM − 1_	None	7 (35%)
	*bla_*ACC*−1_* (AmpC)	1 (5%)
	*bla*_CTX − M−3_ (ESBL)	1 (5%)
	*bla*_CTX − M−65_ (ESBL)/ *bla*_SHV − 12_ (ESBL)	1 (5%)
	*bla*_CTX − M−9_ (ESBL)	1 (5%)
	*bla*_SHV − 12_ (ESBL)	9 (45%)
*bla*_NDM − 5_/*bla*_OXA − 48_	*bla*_CTX − M−14_ (ESBL)/ *bla*_CTX − M−15_ (ESBL)	1 (100%)
*bla*_NDM − 5/_ *bla*_OXA − 181_	*bla*_CMY − 42_ (AmpC)	1 (100%)
*bla*_VIM − 1/_ *bla*_OXA − 48_	*bla*_SHV − 12_ (ESBL)	1 (100%)

### 3.3 Phylogroup assignment and sequence typing

Among the 108 carbapenem-resistant *E. coli* isolates, phylogroup A was the most common (*n* = 40; 37.0%), followed by phylogroup D (*n* = 28; 25.9%), B2 (*n* = 15; 13.9%), and B1 (*n* = 14; 13.0%). Phylogroups E (*n* = 5; 4.6%), C (*n* = 4; 3.7%), and F (*n* = 2; 1.9%) were detected less frequently. The phylogenetic group and sequence type of each analyzed *E. coli* isolate are presented in Supplementary Table S5.

Overall, 67 different STs were detected among the 108 CP–*E. coli* isolates, including two novel STs. The high diversity of STs in relation to carbapenemase genes is shown in the treemap in Figure 2 and distribution of carbapenemase genes across the main *E. coli* sequence types is shown in Supplementary Figure S2.

**Figure 2:**
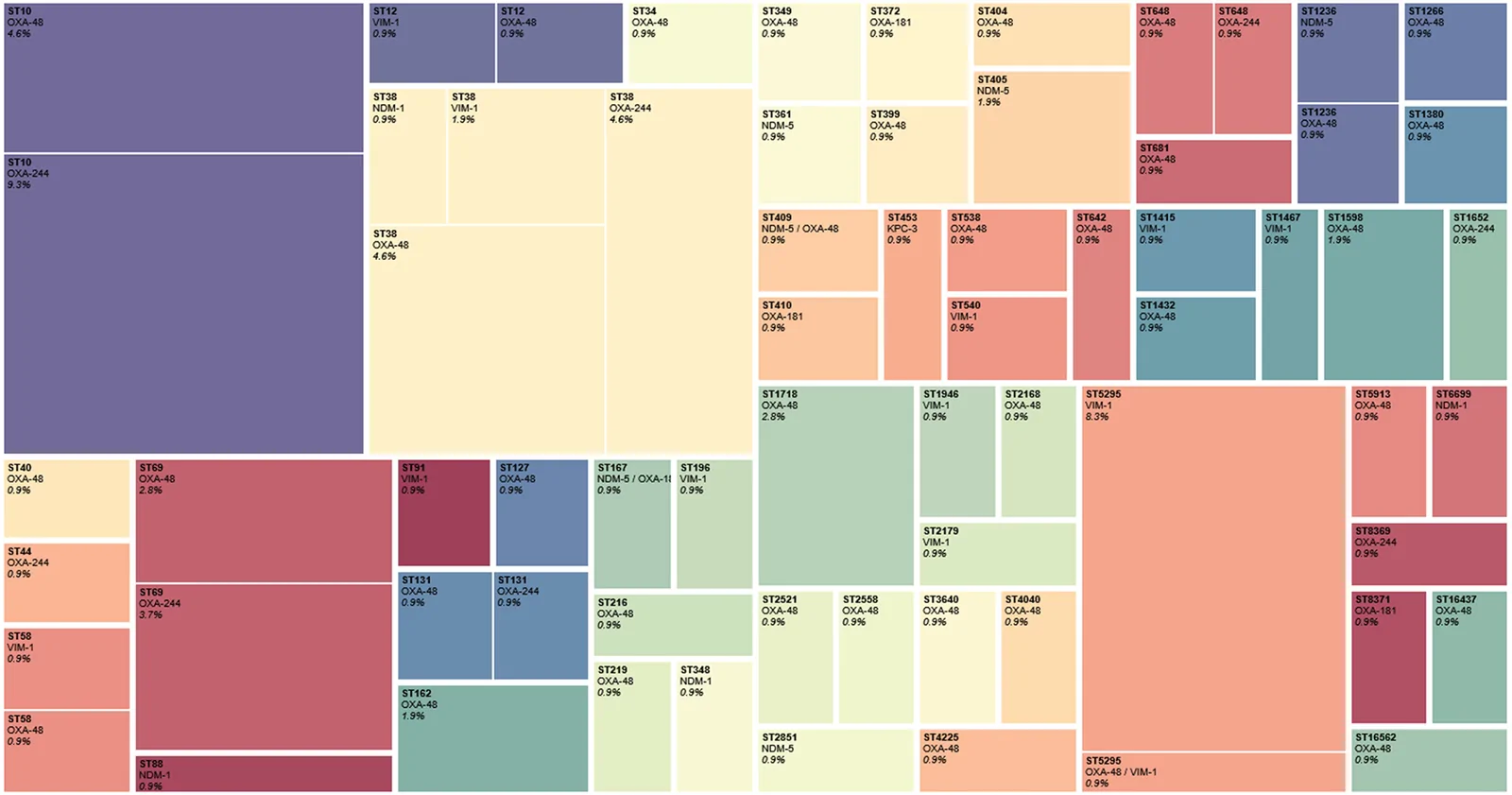
The treemap of CP–*E. coli* sequence types (STs, Warwick scheme) and carbapenemase gene distribution.

The most prevalent lineage in this dataset was ST10 (15 isolates), phylogroup A, which represented the largest subset and was predominantly associated with OXA-244 (10/15) and OXA-48 (5/15) carbapenemases. With the exception of one isolate obtained from a urine sample, all isolates were recovered from surveillance samples.

ST38 (phylogroup D) was the second most represented high-risk lineage in our dataset (13 isolates), harboring diverse carbapenemase genes, including OXA-48 and OXA-244 (five isolates each), VIM-1 (two isolates), and NDM-1 (one isolate). Four isolates were recovered from clinical samples (two tracheal aspirates and two urine samples), while the remaining ST38 isolates were obtained from surveillance samples.

ST5295 (phylogroup A) was the third most frequent ST in the collection (10 isolates), associated with VIM-1 (9 isolates) and OXA-48/VIM (1 isolate). With the exception of one isolate obtained from a urine sample, all ST5295 isolates were recovered from surveillance samples.

ST69 (phylogroup D) was also among more common ST with 7 isolates, associated with either OXA-244 (4 isolates) or OXA-48 (3 isolates). With the exception of one isolate obtained from a blood sample, all ST69 isolates were recovered from surveillance samples.

Other notable lineages were identified only sporadically, including ST131 (2 isolates; one OXA-244-producing isolate and one OXA-48-producing isolate), ST405 (2 isolates; both NDM-5-producing isolates), ST648 (2 isolates; one OXA-48-producing isolate and one OXA-244-producing isolate), ST167 (1 isolate; one NDM/OXA-181-producing isolate), and ST410 (1 isolate; one OXA-181-producing isolate), all originating from surveillance samples. The second isolate from a blood culture belonged to ST1236 (phylogroup B2).

### 3.4 Assessment of genetic relatedness by cgMLST

cgMLST analysis identified six clusters of CP–*E. coli* (Figure 3). MST Cluster 1 was the largest, comprising 10 of 108 isolates, all belonging to ST5295 and carrying either *bla*_VIM − 1_ (9/10) or *bla*_VIM − 1_/*bla*_OXA − 48_ (1/10), indicating clonal spread. VIM-1 carbapenemase was located on an IncA conjugative plasmid. MST Cluster 2 comprised of 7 ST10 (7/108) isolates, also indicating clonal spread with *bla*_OXA − 244_ located on the chromosome. MST Cluster 3 included three ST1718 (3/108) isolates carrying *bla*_OXA − 48_ on an IncL/M type plasmid. Three additional clusters consisting of two isolates were identified: ST38 with *bla*_OXA − 244_, ST69 with *bla*_OXA − 244_, and ST405 with *bla*_NDM − 5_. The remaining isolates (82/108, 75.9%) did not cluster and were considered sporadic.

**Figure 3:**
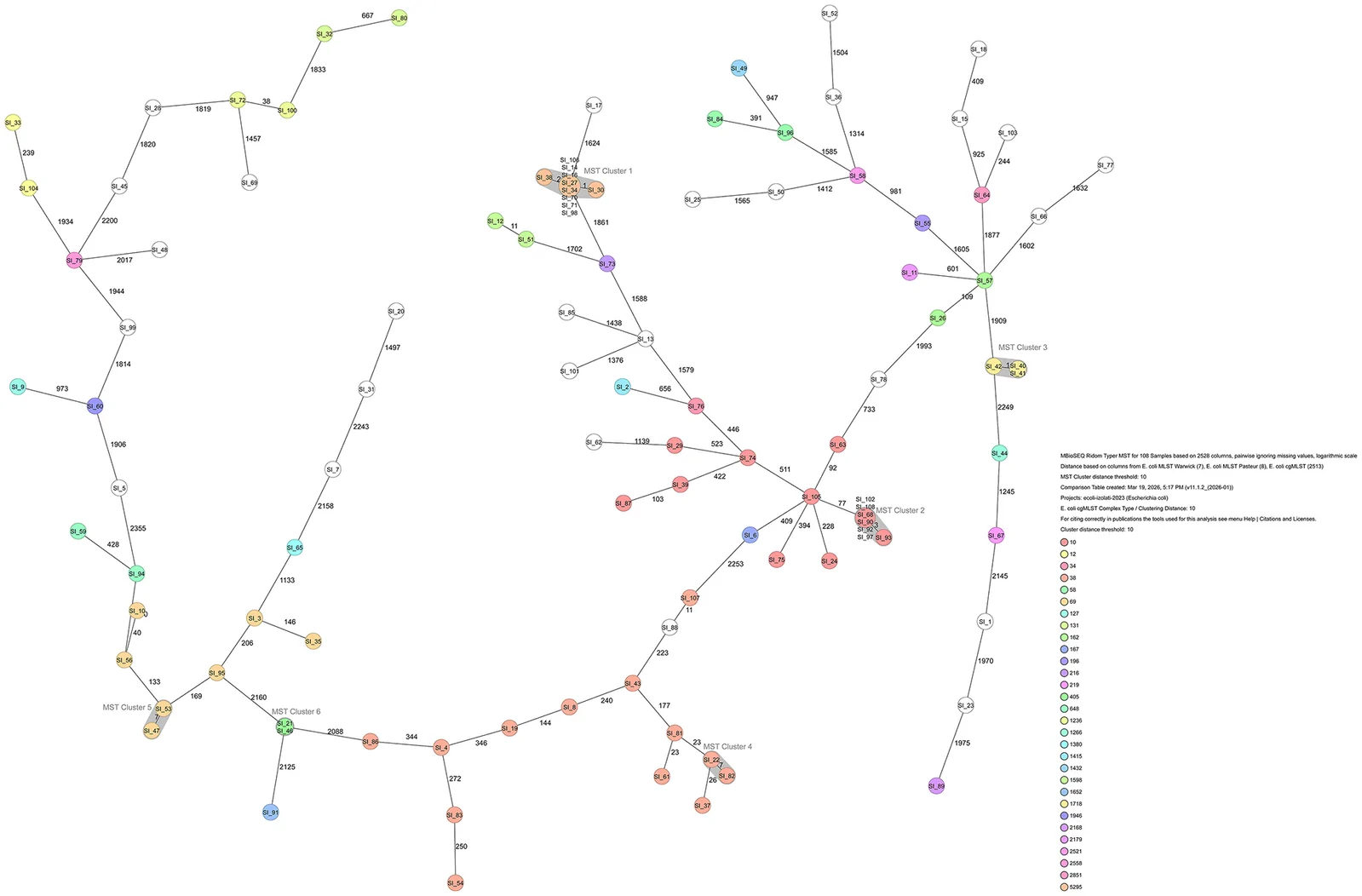
Assessment of genetic relatedness of CP–*E. coli* by cgMLST MBioSeq.

### 3.5 Assessment of plasmid structure

Plasmid analysis based on short-read sequencing data revealed extensive horizontal dissemination of carbapenemase-carrying plasmids (Supplementary Figure S3). Multiple primary MOB-suite clusters comprising at least two plasmids were identified, and 85 of 108 (79%) isolates carried plasmids assigned to shared primary MOB clusters.

As primary MOB clusters group plasmids with approximately 96% nucleotide sequence identity, these findings suggest widespread circulation of closely related plasmid backbones across diverse STs (Figure 4). This was further supported by SNP analysis, which showed only a small number of SNP differences between plasmids within the same cluster, consistent with recent horizontal transfer across genetically distinct isolates (Supplementary Figure S1).

**Figure 4:**
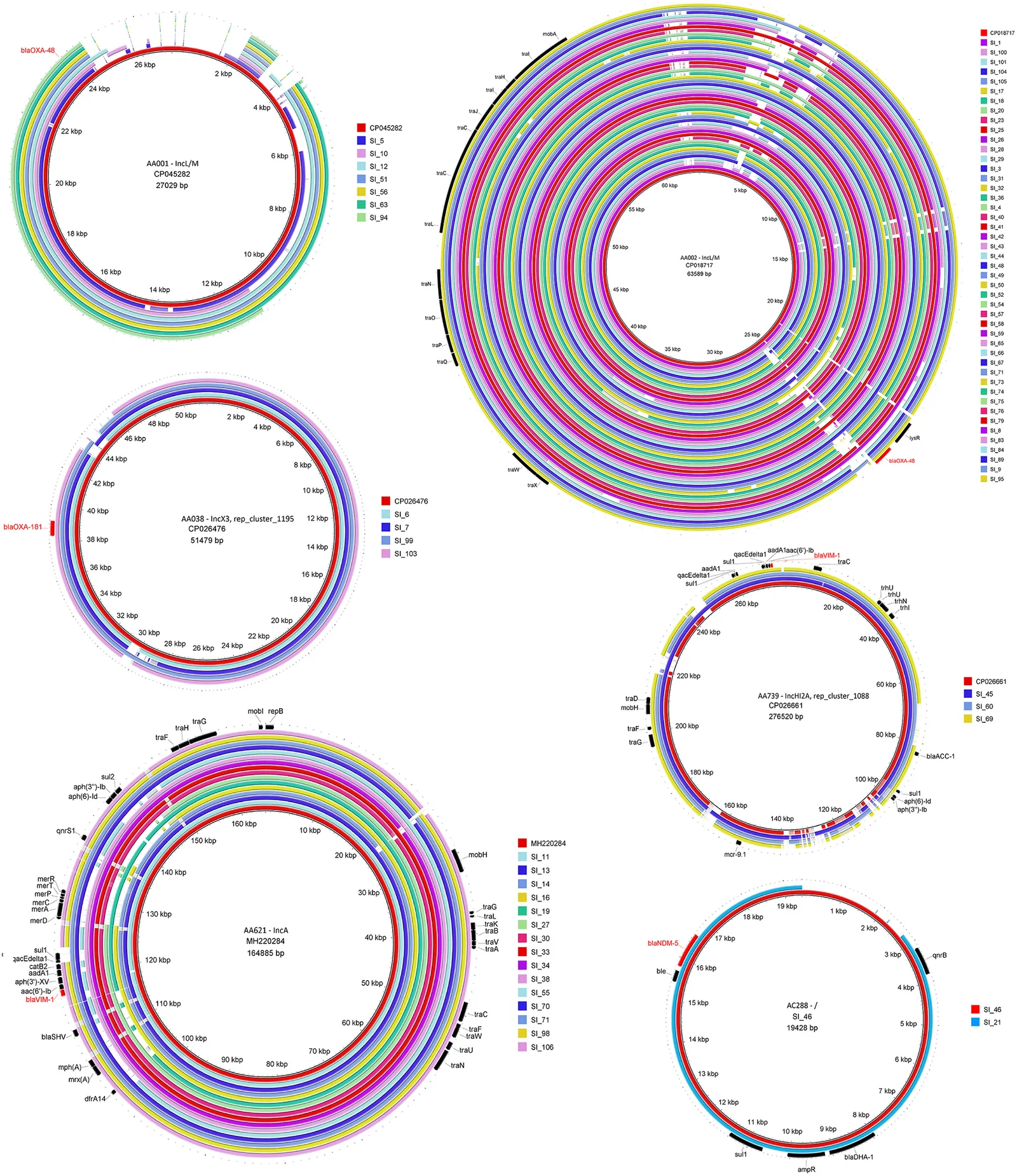
Comparative BRIG analysis of carbapenemase-carrying plasmids within primary MOB-suite clusters. For BRIG visualization, complete plasmid sequences from NCBI were selected as external references based on cluster assignment and sequence similarity, and these reference plasmids are shown within the corresponding BRIG plots. For cluster AC288, the closest NCBI reference plasmid did not contain the *bla*NDM-5 gene; therefore, plasmid SI_46 was selected as reference. Concentric rings represent plasmids assigned to the same primary MOB-suite cluster and are color-coded according to sequence type (ST). Shaded regions indicate sequence similarity relative to the reference plasmid.

The IncL/M plasmid was the most prevalent plasmid type (45/79, 57.0%) and was predominantly associated with *bla*_OXA − 48 − like_ genes (43/45), while two IncL/M plasmids carried *bla*_NDM − 1_. The second most common plasmid type was IncA (14/79, 17.7%), which exclusively harbored blaVIM-1. Additional *bla*_VIM − 1_-carrying plasmids belonged to the IncHI2A and IncN incompatibility groups.

The *bla*_OXA − 48_ gene was strongly associated with IncL/M plasmids, whereas *bla*_VIM − 1_ was primarily located on IncA plasmids. In contrast, *bla*_NDM_ variants were detected on a diverse range of plasmid backbones, including IncC, IncFIA, IncFIB/IncHI1B, IncI-gamma/K1, IncL/M, and IncX3 plasmids. The *bla*_OXA − 181_ gene was predominantly associated with IncX3 plasmids. An overview of plasmid characteristics for each isolate, including the carbapenemase genes located on individual plasmids, is provided in Supplementary Table S6. Detailed MOB-suite results are presented in Supplementary Table S7.

## 4 Discussion

World Health Organization (WHO) has classified resistance to third-generation cephalosporins and carbapenems in Enterobacterales among the critical priorities for the development of new antimicrobial agents (38). The increasing incidence of CP–*E. coli* has been reported worldwide particularly over the past decade, and is frequently associated with the dissemination of a limited number of successful high-risk clonal lineages carrying carbapenemase genes (9, 10).

To assess the burden and dissemination of CPE in Europe, three structured European surveys have been conducted to date (9, 13, 14). In 2020, the EURGen-RefLabCap project was initiated to strengthen genomic surveillance and reference laboratory capacity for AMR across 37 European countries (16). Slovenia has participated in all three structured European surveys and was one of the 13 priority countries identified through a mapping exercise. Overall, Slovenian data show that while CP–*E. coli* is detected less frequently than CP–*K. pneumoniae*, its incidence is increasing. To enable comparisons across national surveys undertaken as part of structured European surveys, we compared the incidence of clinical CP–*E. coli* isolates, as recommendations and practices for surveillance of CPE Enterobacterales have varied over time and between hospitals. In the 2013–2014 EuSCAPE survey no clinical CP–*E. coli* isolates were detected in Slovenia during the 6-month study period. In the 2019 CCRE survey, six CP–*E. coli* isolates were identified, comprising three OXA-48 producers (ST38, ST224, and ST973), two KPC-3 producers (both ST131), and one NDM-5 producer (ST410); however, only one isolate, a KPC-producing ST131 strain, originated from a clinical sample during the 6-month study period (9, 14). In contrast, the EURGen-RefLabCap pilot project included all CP–*E. coli* isolates recovered from Slovenian patients during 2023 (1-year period) and a total of 12 patients had isolates from clinical samples, including two from blood cultures. Available Slovenian national data, derived from surveillance activities and studies performed by the Slovenian Society for Clinical Microbiology and Hospital Infection and the Slovenian NAC, have also demonstrated a substantial increase in CP–*E. coli* over the period 2017–2025 as the number of patients with CP–*E. coli* identified from clinical specimens increased from 2 to 39 (19.5-fold increase). When clinical and surveillance isolates were combined, the number of patients with CP–*E. coli* isolates increased from 10 to 228 (22.8-fold increase), with more pronounced rise observed from 2021 onwards (15). Although differences in study design and surveillance periods should be considered, these findings indicate a considerable increase in the incidence of clinical CP–*E. coli* isolates in Slovenian patients over the past decade. The majority (97/108, 89.8%) of the isolates from the EURGen-RefLabCap pilot project were recovered from surveillance samples, indicating the existence of a larger reservoir of CP–*E. coli* in the Slovenian population. Additionally, a substantial proportion (30.6%) were detected more than 48 h after admission. However, these findings should be interpreted with caution, as recommendations and practices for surveillance of CPE Enterobacterales vary between Slovenian hospitals and over time.

The majority (75/108, 69.4%) of CP–*E. coli* isolates in the EURGen-RefLabCap pilot project collection were phenotypically susceptible to carbapenems, consistent with findings reported by the ECDC at the European level (39, 40). We observed a predominance of serine carbapenemases, mainly OXA-48-like (70.4%), in our collection, while the remaining isolates produced metallo-beta lactamases (26.9%) or co-produced OXA-48-like and a metallo-beta lactamase (2.7%). In contrast, the results of a recently published CCRE survey reported a considerably lower proportion of OXA-48-like carbapenemases (35.7%) and a higher proportion of NDM or VIM carbapenemases than in our collection (48.4%) (9).

Our collection showed marked ST diversity, consistent with the known epidemiology of CP–*E. coli* as a diverse population driven by both horizontal gene transfer and expansion of high-risk lineages (9, 10, 41). Most of high-risk clonal lineages were detected in our collection, the most prevalent was ST10, followed by ST38. Other epidemic lineages were sporadic, including ST131, ST167, ST405, ST410 and ST648 (9, 10). The third most common ST was ST5295 which is a less common, extraintestinal pathogenic *E. coli*. Another relatively common ST in our collection was ST69, a globally disseminated extraintestinal pathogenic *E. coli* that was associated with OXA-48-like carbapenemases in our collection, which is a less common occurrence (9, 10). Phylogroup A was the most prevalent, followed by D, B2, and B1. This distribution indicates that carbapenemase genes were present in both commensal-associated genetic backgrounds, particularly phylogroups A and B1, and in phylogroups D and B2, which include many extraintestinal pathogenic lineages. A similar phylogroup distribution among CP–*E. coli* was reported in a recent French national surveillance study (42). Overall, the broad ST and phylogroup distribution supports dissemination of carbapenemase determinants across multiple *E. coli* genetic backgrounds rather than their restriction to a few dominant lineages.

According to cgMLST analysis, clonal spread occurred in approximately one quarter of isolates (26/108, 24.1%) with six distinct clusters of CP–*E. coli*, most involving patients from two or more Slovenian regions. Additionally, extensive horizontal dissemination of carbapenemase-carrying plasmids was observed. Carbapenemase genes were detected on a diverse range of plasmid types, including IncL/M, IncA, IncF-family, IncHI2A, IncN, and IncI-gamma/K1 plasmids; in one cluster the carbapenemase genes (*bla*_OXA − 244_) were integrated into the chromosome. The predominance of *bla*_OXA − 48_ on IncL/M plasmids and *bla*_VIM − 1_ on IncA plasmids indicates that dissemination of these resistance determinants is primarily driven by successful plasmid lineages capable of spreading across genetically distinct CP–*E. coli* isolates, consistent with the well-established role of horizontal plasmid transfer in their epidemiology (9, 40, 43, 44). The main limitation of this part of the study is that we had only short-read WGS data available which limited the reliability of the plasmid structure reconstructions by MOB-suite. Reconstruction accuracy depends on the availability of related plasmids in reference databases and may be reduced for novel or poorly characterized plasmids. Furthermore, contigs containing mobile genetic elements or sequences shared between plasmids and chromosomes may be incorrectly assigned, resulting in erroneous placement of contigs on either plasmid or chromosome and consequently affecting the inferred genomic location of antimicrobial resistance genes. Therefore, plasmid structures inferred by MOB-suite should be regarded as putative reconstructions and, where possible, validated using long-read or hybrid sequencing approaches.

## 5 Conclusion

The first national study provides a comprehensive genomic overview of CP–*E. coli* in Slovenia within the EURGen-RefLabCap framework. Our findings demonstrate an increasing incidence of clinical CP -*E. coli* isolates in Slovenian patients over the past decade, alongside a substantial reservoir of colonized individuals. The 2023 CP–*E. coli* population is characterized by marked ST diversity with dissemination of carbapenemases driven by both clonal expansion and horizontal gene transfer. However, the widespread occurrence of closely related plasmids across diverse STs suggests that plasmid-mediated transmission played the dominant role. Notably, OXA-48-like and VIM-1 carbapenemases were predominately associated with successful plasmid backbones, underscoring the dominant role of plasmid-mediated transmission in shaping the local epidemiology of CP–*E. coli*. Overall, these results highlight the importance of integrated genomic surveillance to monitor both plasmid dynamics and clonal expansion, in order to better understand and control the evolving epidemiology of CP–*E. coli* and better support targeted infection prevention and control strategies.

## Data Availability

The datasets presented in this study can be found in online repositories. The names of the repository/repositories and accession number(s) can be found below: https://www.ebi.ac.uk/ena, PRJEB115612.

## Supplementary material

Supplementary Figure S1 SNP-based phylogenetic trees of plasmids from three representative primary MOB-suite clusters; AA001, AA002 and AA621. Plasmids within each primary MOB cluster share approximately 96% nucleotide sequence identity. The trees illustrate the SNP-based genetic relationships among plasmids within each cluster, with branch lengths proportional to the number of SNP differences.

Supplementary Figure S2 Phylogenetic tree based on the concatenated sequences of the seven Warwick MLST loci showing the distribution of carbapenemase genes across the main *E. coli* sequence types.

Supplementary Figure S3 Sankey diagram showing the distribution of carbapenemase genes among primary MOB plasmid clusters and Warwick sequence types of *E. coli* isolates.

Supplementary Table S1 Quality-control metrics for whole-genome sequencing data and genome assemblies, with corresponding ENA accession numbers.

Supplementary Table S2 Alignment statistics for plasmids assigned to primary MOB-suite clusters and summary of statistics for the core SNP alignment.

Supplementary Table S3 Reference plasmids used for SNP analysis (Table A) and comparative visualization with BRIG (Table B).

Supplementary Table S4 Antimicrobial resistance determinants detected in *E. coli* isolates using AMRFinderPlus.

Supplementary Table S5 Phylogenetic group and sequence type distribution of *E. coli* isolates.

Supplementary Table S6 Plasmid characteristics of *E. coli* isolates.

Supplementary Table S7 Plasmid-associated elements identified in *E. coli* isolates using MOB-suite.
